# Investigating recovery after a spontaneous intracerebral haemorrhage in zebrafish larvae

**DOI:** 10.1093/braincomms/fcae310

**Published:** 2024-09-12

**Authors:** Siobhan Crilly, Isabel Shand, Abigail Bennington, Emily McMahon, Daisy Flatman, Victor S Tapia, Paul R Kasher

**Affiliations:** Division of Neuroscience, School of Biological Sciences, Faculty of Biology, Medicine and Health, Manchester Academic Health Science Centre, The University of Manchester, Manchester M13 9PT, UK; Geoffrey Jefferson Brain Research Centre, The Manchester Academic Health Science Centre, Northern Care Alliance and University of Manchester, Manchester M13 9PT, UK; Division of Neuroscience, School of Biological Sciences, Faculty of Biology, Medicine and Health, Manchester Academic Health Science Centre, The University of Manchester, Manchester M13 9PT, UK; Geoffrey Jefferson Brain Research Centre, The Manchester Academic Health Science Centre, Northern Care Alliance and University of Manchester, Manchester M13 9PT, UK; Division of Cardiovascular Sciences, School of Medical Sciences, Faculty of Biology, Medicine and Health, Manchester Academic Health Science Centre, The University of Manchester, Manchester M13 9PT, UK; Division of Neuroscience, School of Biological Sciences, Faculty of Biology, Medicine and Health, Manchester Academic Health Science Centre, The University of Manchester, Manchester M13 9PT, UK; Geoffrey Jefferson Brain Research Centre, The Manchester Academic Health Science Centre, Northern Care Alliance and University of Manchester, Manchester M13 9PT, UK; Division of Neuroscience, School of Biological Sciences, Faculty of Biology, Medicine and Health, Manchester Academic Health Science Centre, The University of Manchester, Manchester M13 9PT, UK; Geoffrey Jefferson Brain Research Centre, The Manchester Academic Health Science Centre, Northern Care Alliance and University of Manchester, Manchester M13 9PT, UK; Division of Neuroscience, School of Biological Sciences, Faculty of Biology, Medicine and Health, Manchester Academic Health Science Centre, The University of Manchester, Manchester M13 9PT, UK; Geoffrey Jefferson Brain Research Centre, The Manchester Academic Health Science Centre, Northern Care Alliance and University of Manchester, Manchester M13 9PT, UK; Division of Neuroscience, School of Biological Sciences, Faculty of Biology, Medicine and Health, Manchester Academic Health Science Centre, The University of Manchester, Manchester M13 9PT, UK; Geoffrey Jefferson Brain Research Centre, The Manchester Academic Health Science Centre, Northern Care Alliance and University of Manchester, Manchester M13 9PT, UK

**Keywords:** stroke, intracerebral haemorrhage, rehabilitation, recovery, neural progenitor cells

## Abstract

Intracerebral haemorrhage is a debilitating stroke sub-type with high morbidity and mortality rates. For survivors, rehabilitation is a long process, and with no available therapeutics to limit the immediate pathophysiology of the haemorrhage, recovery is dependent on individual neuroplasticity. We have previously shown that zebrafish larvae can be used to model spontaneous brain haemorrhage. Zebrafish exhibit innate recovery mechanisms and are often used as a model system for investigation into regeneration after injury, including injury to the nervous system. Here, we investigate the spontaneous and immediate recovery in zebrafish larvae following an intracerebral haemorrhage at 2 days post-fertilisation, during pre-protected stages and over the first 3 weeks of life. We have shown that following the onset of bleed at ∼2 days post-fertilisation zebrafish are capable of clearing the haematoma through the ventricles. Brain cell damage associated with intracerebral haemorrhage is resolved within 48 h, and this recovery is associated with survival rates equal to wildtype and non-haemorrhaged sibling control animals. Larvae express more nestin-positive neural progenitor cells 24 h after injury when the most damage is observed, and through mass spectrometry analysis, we have determined that these cells are highly proliferative and may specially differentiate into oligodendrocytes. This study provides an insight into the haematoma resolution processes in a live, intact organism, and may suggest potential therapeutic approaches to support the recovery of intracerebral haemorrhage patients.

## Graphical Abstract

**Graphical Abstract:**
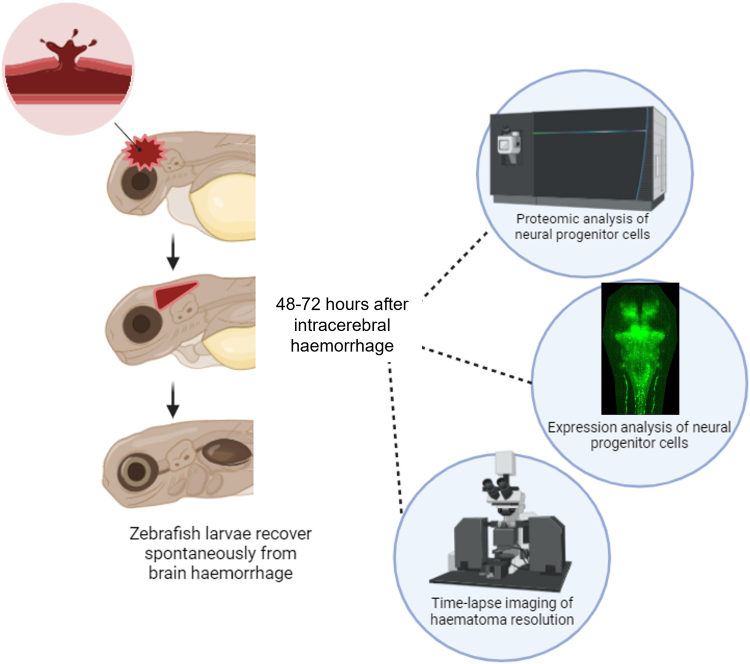

## Teaser

In this study, Crilly *et al* utilised a larval zebrafish model of intracerebral haemorrhage to exploit the innate regenerative properties of the species and investigate the spontaneous resolution of haematoma and the role of neural progenitor cells to identify mechanisms that could contribute to clinical recovery.

## Introduction

Intracerebral haemorrhage (ICH) is a severe sub-type of stroke with a high rate of morbidity and mortality. The high disability burden associated with ICH^1^ means that understanding recovery and rehabilitation to reveal novel therapeutic strategies will have a broad clinical impact. The majority of clinical research into ameliorating disability in stroke survivors has been to re-establish independence in activities of daily living.^2^ Timing, intensity, and schedule are important paradigms that influence the optimal rehabilitation for patients about which, little is known. Pre-clinical studies have revealed that early rehabilitation methods have improved outcomes in mammals (one week post-ICH)^3-5^ and have been reviewed elsewhere.^6^ Mechanisms underlying behavioural recovery in mammals are not fully understood but have been associated with increased dendritic spine density, neuronal progenitor cell migration, neurogenesis and increased bone-derived neurotrophic factor signalling.^7,8^ Regenerative processes such as cell proliferation, differentiation, myelination and angiogenesis occur in animal models of ICH.^9^ Mesenchymal stem cell (MSC) transplantation research for ICH recovery has gained traction in recent years. Many efforts have been made to find the optimal delivery method and mix of MSC and MSC release components for therapy, to promote neurogenesis and angiogenesis while inhibiting deleterious inflammatory and apoptotic processes.^10^ Research into how to better progress recovery after ICH is still inconclusive.

We have previously shown that bubblehead zebrafish larvae, with a hypomorphic mutation in βpix (*arhgef7*), can be used to model ICH^11,12^ and make translationally relevant discoveries for the treatment of ICH.^13^ The reduced function of βpix inhibits cytoskeleton remodelling in the developing neurovasculature and endothelial cells are detached from the underlying mesenchyme and thus vulnerable to vessel rupture. Zebrafish also retain remarkable tissue regenerative properties into adulthood and have been used to model recovery from injury to the heart, central nervous system, pancreas, liver, kidney and other peripheral tissues.^14^ In this study, we sought to apply the zebrafish model of spontaneous ICH to investigate the innate recovery timeline and cellular processes that may contribute to spontaneous resolution of the haematoma.

Clinical resolution of a cerebral haematoma is dependent on location, size and whether surgical evacuation was performed however, a haematoma may be visible by CT scan for between 6 months to a year.^15^ In mammals, literature suggests erythrocytes may be cleared from the haematoma through active phagocytosis by macrophages/microglia, migration to the lymphatic system, or through lysis due to complement binding or metabolic depletion.^16,17^ Understanding these processes to accelerate the reduction of the haematoma will have beneficial outcomes for patients.^18^ During mammalian neurodevelopment, neurons are generated from asymmetric division of neural progenitor cells (NPC). NPC multi-potency means that they can give rise to both mature post-mitotic neurons and glial cells that are essential for the recovery of brain function.^9^ Nestin is a stem cell marker expressed in proliferating precursor cell populations in the developing embryonic, and in restricted regions of the post-embryonic, central nervous system.^19^ Investigations in mouse ischaemic stroke models have revealed an increased localized nestin expression at the infarct site.^20^ In experimental ICH, rat models exhibited increased nestin expression in astrocytes in the week following haemorrhage.^21^ In this study, we sought to characterize the nestin positive NPCs in zebrafish to identify potential new mechanisms that influence proliferation, migration and differentiation in the ICH recovery timeline.

## Materials and methods

### Zebrafish husbandry

Zebrafish (*Danio rerio*) were raised and maintained at The University of Manchester Biological Services Unit under standard conditions as previously described.^22^ Adult zebrafish husbandry was approved by The University of Manchester Animal Welfare and Ethical Review Board. All experiments were performed in accordance with the UK Home Office regulations (PPL: P132EB6D7 and PPL: PP1968962) and reported according to the ARRIVE guidelines.^23^ Transgenic zebrafish used for this study include *ubiq*:secAnnexinV-mVenus, a fluorescent reporter for cell death,^24^ neural stem cell marker Tg(*nestin*:GFP),^19^ erythroid-specific Tg(*gata1:*dsRed)^25^ and an endothelial cell-specific Tg(*fli1*:EGFP)(y1)^26^ (herein annexinV, nestin and fli1:gata, respectively) on a mutant *bubblehead* (herein *bbh*) (*bbh*^m292^),^27^ nacre (*mitfa*^w2/w2^) or wildtype (AB Nott) background. Fertilized embryos were collected from natural spawning and incubated at 28°C in standard E3 medium and staged according to standard guidelines. Zebrafish larvae used for experimentation were terminated prior to protected status with an overdose of 4% MS222 anaesthesia and freezing at −20°C. Larvae with protected status were Schedule 1 terminated with an overdose of AquaSed anaesthesia and confirmation of death with freezing at −80°C.

### Study groups

For the bbh line,^27^ embryos were obtained from adult in-crosses of heterozygous *bbh*^m292^ mutant animals, on both wildtype and transgenic reporter backgrounds. Embryos with haemorrhages (ICH+) were separated from non-haemorrhaged (ICH−) siblings at ∼52 h post-fertilisation (hpf) for downstream analyses. Sample groups are per clutch of eggs unless stated otherwise.

### Kaplan–Meier analysis

Sibling larvae were separated for ICH− and ICH+. Wildtype fish were bred at the same time and the larvae were used as age matched controls. Larvae were randomly selected at pre-sex stages (*n* = 30 per group), and were raised under standard conditions, including in a rotifer polyculture from 5 to 10 days post-fertilisation (dpf).^28^ Fish were counted daily for 21 days and survival rates recorded. Animals were terminated at experiment end with an overdose of 4% MS222 anaesthesia and freezing at −80°C.

### Imaging

Prior to imaging, larvae were anaesthetized using 0.02% MS222. Live imaging of ICH+ and ICH− fish occurred repeatedly using a Leica S9e with a Kern Microscope VIS 2.0Pro and software, fluorescent images were acquired using a Leica M165FC fluorescent stereomicroscope and processed using Las-X (version 3.3.3.16958). For lightsheet imaging, larvae were suspended in 1.5% low-melt agarose within a chamber that was filled with 0.02% MS222 in E3 embryo water without methylene blue. Lightsheet images were acquired using a Zeiss Z.1 Lightsheet equipped with a W Plan-Apochromat 20 × magnification/1.0 UV–vis objective and processed with ZEN imaging software (version 2.3). Time-lapse videos were constructed from successive z-stack images acquired every 5 min for 3 h.

### Nestin intensity fluorescence analysis

Images acquired by lightsheet microscopy at 2, 3, 4 and 5 dpf were analysed using ImageJ and Inkscape. In brief, brain regions were manually selected to exclude any fluorescence from the eyes and divided down the midline of the brain and at a 90° angle across the mid/hind brain boundary. Intensity fluorescence in selected regions and in overall brain areas was determined.

### IMARIS image analysis

Z-stack images of ICH+ larvae acquired on the lightsheet were compressed into sub-sets of 50 z images. IMARIS software (Oxford Instruments, version 10) was used to generate volume measurements of haemorrhages. In brief, researcher was blinded to image origin group, areas of interest were selected surrounding the haematoma and the ‘surfaces’ tool used to determine volume. Areas were thresholded to absolute intensity, and the minimum number of voxels included adjusted to eliminate the selection of single red blood cells (RBCs).

### Dextran injections

At 27 hpf, nestin larvae on bbh and wild-type backgrounds were incubated in water containing 1-phenol 2-thiourea to prevent the formation of pigment. At 2 dpf, larvae were anaesthetized in 0.02% MS222 and 1 nL of 50 µg/mL dextran Texas Red (molecular weight 70 kDa) was injected into the hind-brain ventricle. Larvae were imaged using a Leica M165FC fluorescent stereomicroscope.

### *o*-Dianisidine staining and bleaching

Dechorionated zebrafish larvae were incubated in the dark with 500 μL of *o*-dianisidine solution as previously described.^29^ Larvae were fixed in 4% PFA overnight at 4°C before rinsing with 0.1% PBS-Tween and pigment was bleached using 10% hydrogen peroxide (5% formamide, 0.5 × SSC). A total of 123 bbh larvae (ICH− and ICH+) were stained from 2 to 5 dpf and visualized under a light microscope (Leica S9e). Reference images for identifying brain regions were from mapzebrain.org (www.mapzebrain.org/atlas/2d), the Max Planck zebrafish brain atlas resource.

### Fluorescence-associated cell sorting

Nestin positive larvae were grouped for ICH+ and ICH− at 3 and 4 dpf and heads removed into ice-cold PBS (*n* = 35 larvae for each group). Cells were dissociated with equal volumes of collagenase/dispase (10 µg/mL) and TrypLE (Thermo Fisher) at 28°C for 1 h. DNaseI (Invitrogen) was added to the single cell suspension for 30 min, samples spun on maximum speed, and supernatant removed. The pellet was resuspended in 20 mM EDTA and 10% normal goat serum and filtered. Nestin-positive cells were sorted using the BD Influx cell sorter to obtain 100 000 cells (3 dpf) and 250 000 cells (4 dpf) per group.

### Liquid chromatography mass spectrometry

Nestin positive cells isolated by FACS were lysed in S-Trap buffer. Proteins were reduced, alkylated and digested using the standard S-Trap column protocol and trypsin digestion at 2 µg/µL. Peptides were eluted into a solution of 30% aqueous acetonitrile with 0.1% formic acid. Peptides were desalted with POROS R3 beads and eluted into a final solution of 30% aqueous acetonitrile with 0.1% formic acid. Samples were completely dried using a Heto speed vacuum centrifuge.

LC-MS/MS was carried out by the University of Manchester BioMS core facility and separation was performed on a Thermo RSLC system consisting of a NCP3200RS nano pump, WPS3000TPS autosampler and TCC3000RS column oven configured with buffer A as 0.1% formic acid in water and buffer B as 0.1% formic acid in acetonitrile. An injection volume of 2 µL was loaded into the end of a 5 µL loop and reverse flushed onto the analytical column (Waters nanoEase M/Z Peptide CSH C18 Column, 130Å, 1.7 µm, 75 µm × 250 mm) kept at 35°C at a flow rate of 300 nL/min for 8 min, with an initial pulse of 500 nL/min for 0.3 min to rapidly re-pressurize the column. The injection valve was set to load before a separation consisting of a multi-stage gradient of 1% B to 6% B over 2 min, 6% B to 18% B over 44 min, 18% B to 29% B over 7 min and 29% B to 65% B over 1 min before washing for 4 min at 65% B and dropping down to 2% B in 1 min. The complete method time was 75 min. The analytical column was connected to a Thermo Exploris 480 mass spectrometry system via a Thermo nanospray Flex Ion source via a 20 µm ID fused silica capillary. The capillary was connected to a fused silica spray tip with an outer diameter of 360 µm, an inner diameter of 20 µm, a tip orifice of 10 µm and a length of 63.5 mm (New Objective Silica Tip FS360-20-10-N-20-6.35CT) via a butt-to-butt connection in a steel union using a custom made gold frit (Agar Scientific AGG2440A) to provide the electrical connection. The nanospray voltage was set at 1900V and the ion transfer tube temperature set to 275°C.

Data were acquired in a data dependent manner using a fixed cycle time of 1.5 s, an expected peak width of 15 s and a default charge state of 2. Full MS data were acquired in positive mode over a scan range of 300–1750 Th, with a resolution of 120 000, normalized AGC target of 300% and a max fill time of 25 mS for a single microscan. Fragmentation data were obtained from signals with a charge state of +2 or +3 and an intensity over 5000 and they were dynamically excluded from further analysis for a period of 15 s after a single acquisition within a 10 ppm window. Fragmentation spectra were acquired with a resolution of 15 000 with a normalized collision energy of 30%, a normalized AGC target of 300%, first mass of 110 Th and a max fill time of 25 mS for a single microscan. All data were collected in profile mode.

Analysis was performed using Proteome Discoverer (PD) (v2.5). The data were processed using the consensus workflow provided with PD in the file CWF_Comprehensive_Enhanced_Annotation_LFQ_and_Precursor_Quan and the processing workflow provided with PD in the file PWF_QE_Precursor_Quan_and_LFQ_SequestHT_Percolator. The processing workflow was set to search the SwissProt Danio rerio protein database (v2023-05-09) and TrEMBL *Danio rerio* protein database (v2023-05-09) (https://www.uniprot.org/) using the Sequest protein identification search engine algorithm provided with PD. The protein identification algorithm was set to trypsin cleaving at lysine and arginine. Two missed cleavage events were permitted. The algorithm searched for a fixed modification of carbamidomethyl (+57.021 Da) to cysteines and a dynamic modification of oxidation (+15.995 Da) to methionine. A precursor tolerance of 10 ppm and a fragmentation tolerance of 0.02 Da was used. A false discovery rate (FDR) was calculated for both the protein level and the peptide level by PD. Proteins were labelled with high confidence where the FDR was <0.01, medium confidence where the FDR was between 0.01 and 0.05, and low confidence where the FDR was >0.05.

To determine effect of haemorrhage, data ratios were determined for 3 dpf ICH+/ICH− and 4 dpf ICH+/ICH−. Proteins that were significantly up/downregulated (−1.0 > log2 < 1.0) were assessed for pathway enrichment using FishEnrichr (https://maayanlab.cloud/FishEnrichr/) and the Kyoto Encyclopedia of Genes and Genomes database (2023).

### Western blot

Zebrafish larval heads were removed into ice-cold PBS, using a bevelled needle. Cells were dissociated using a 1% Triton X lysis buffer and protease inhibitor. 300 µg of protein was loaded into resolving gels (8% for nestin, 12% for neuN and pcna, 15% for olig2) and separated before blotting using a semi-dry transfer system (BIO-Rad). Primary antibodies (rabbit anti-PCNA: GeneTex, GTX124496, rabbit anti-nestin: Cell Signalling, 10959S,mouse anti-NeuN: Antibodies.com, A85405 and rabbit anti-Olig2: Abcam, ab109186) were added 1:1000 in 1% BSA overnight at 4°C and species specific secondary HRP antibodies (Agilent Technologies) (1:5000) used to visualize bands with ECL detecting reagent (Amersham) in the G:Box with GeneSys software (Syngene). Membranes were washed and incubated with anti-β-actin (Sigma A3854) 1:20 000 as a loading control. Gel bands were quantified using the ImageJ gel analysis tool, measuring the area under the curve for each band intensity.

### Statistical analysis

Samples sizes were determined by preliminary data using *α*= 0.05 and *β* = 0.80. Data were analysed for statistical significance on GraphPad Prism (version 9.5) and displayed as mean ± standard deviation (SD) unless stated otherwise. Data acquired for number, volume, surface area and SA:V ratio of ICH were analysed using a repeated measures One-way ANOVA with uncorrected Fishers comparisons between days as data has no multiple comparisons. To determine differences in intensity of nestin fluorescence, a one-way ANOVA with uncorrected Fishers comparison where no multiple comparisons were required and Tukey *post hoc* test was used and comparisons made within time groups. *F* values are presented with degrees of freedom (DF) numerator, DF denominator.

## Results

### Brain haemorrhages in homozygous bbh larvae are varied in location and size that resolve in pre-protected stages

**Figure 1:**
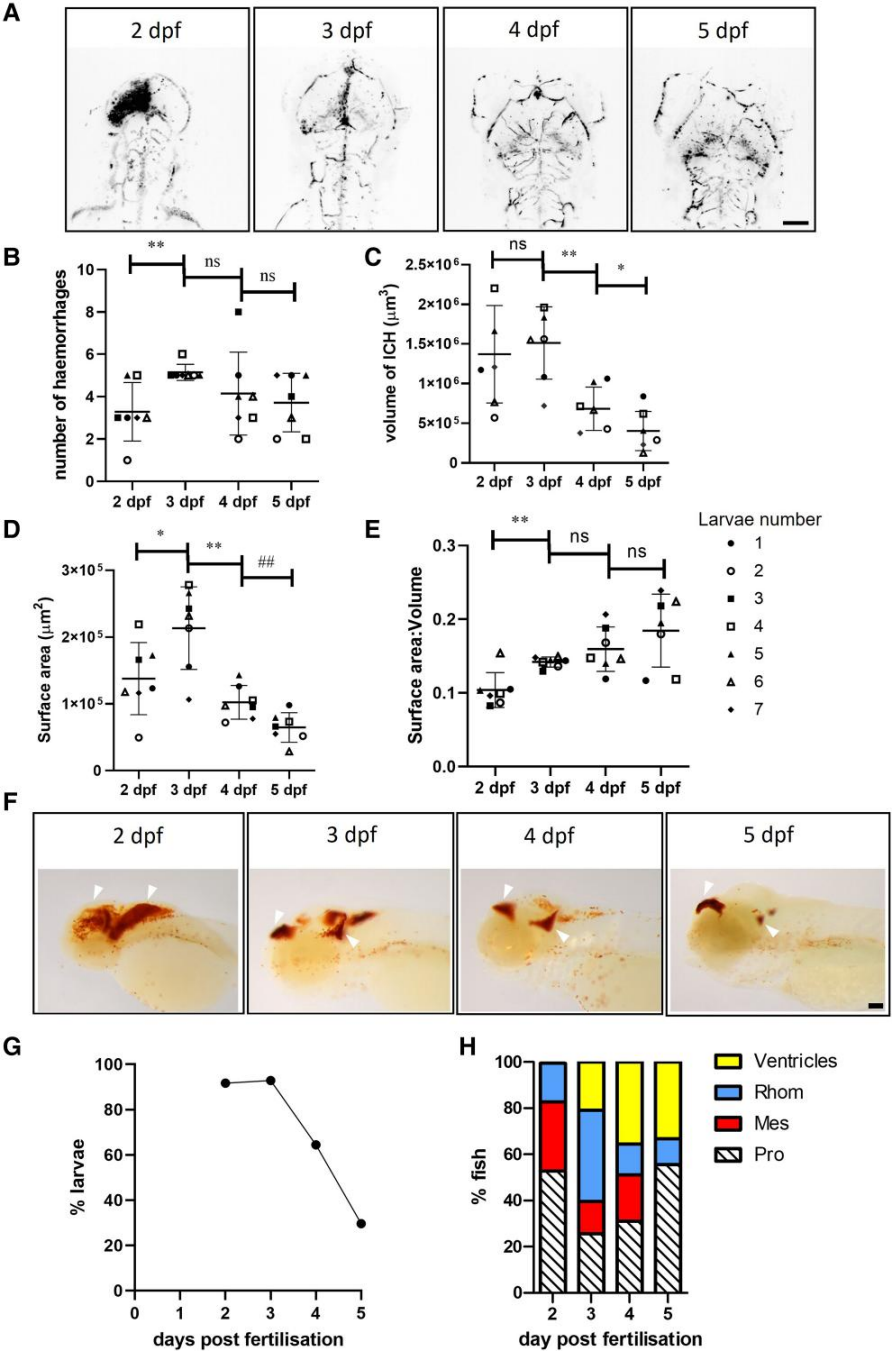
**Larvae exhibit variable locations and sizes of haematoma, and resolution is spontaneous.** (**A**) Representative dorsal view, inverted images of gata1:dsRed fluorescence in a single larva (Fish 7) from 2, 3, 4 and 5 dpf showing haematoma clearance and restoration of circulation. Scale bar = 100 µm. (**B**) The number of haemorrhages in ICH+ larvae over time (*n* = 7) **P = 0.0068, *F*(2.290, 13.74) 2.638 (**C**) the volume of the haemorrhages **P = 0.0042 *P = 0.0119, *F*(2.047, 12.28) = 15.23. (**D**) The surface area of the haemorrhage *P = 0.012, **P = 0.0023, ##*P* = 0.0024, *F*(1.934, 11.60) = 21.27 and (**E**) the surface area to volume ratio of the haemorrhage **P = 0.002, *F*(1.711, 10.27) = 9.793. Symbol points pair time data for each individual larva. Data were analysed using a repeated measures one-way ANOVA with uncorrected Fishers *post hoc* analysis to account for no multiple comparisons (**F**) Representative images of larvae stained for haemoglobin at 2 (*n* = 24), 3 (*n* = 28), 4 (*n* = 45) and 5 (*n* = 27) dpf shows the distribution of haematoma across the brain changes over time (white arrows). Scale bar = 75 µm. (**G**) Percentage of larvae with visible haemorrhage over time. Analysis generated from data in (**F**). (**H**) Location of bleeds over time as scored from larvae in (**F**). dpf, days post-fertilisation; ICH, intracerebral haemorrhage; ns, non-significant; Pro, prosencephalon; mes, mesencephalon; rhom, rhombencephalon.

Homozygous bbh mutants spontaneously haemorrhage in the brain after ∼33 hpf.^11,27^ These haemorrhages are associated with increased brain cell death and impaired larval motility when compared to ICH− siblings, as described previously.^11,12^ Here, we sought to determine whether haemorrhages occur in homogenous brain regions, numbers or sizes between individual larvae. Individual gata1 positive larvae (*n* = 7) were imaged at 2, 3, 4 and 5 dpf to visualize the haematoma (Fig. 1A). Expansive haemorrhages at 2 dpf were observed across the brain, which appear condensed to ventricular spaces at 3 dpf with gata1 fluorescence more diffuse, possibly attributed to the death and breakdown of RBCs. Normal brain circulation appeared to be restored at 4 and 5 days. The average number of haemorrhages per larvae at 2 dpf was 3.3, which on average increased over time as haematomas were broken down (Fig. 1B). Z-stack images were 3D rendered to determine the volume of blood (Fig. 1C) and the surface area of the haematoma(s) (Fig. 1D), both of which decreased over the recovery period. The average surface area to volume ratio (SA:V) increased over time, indicating that as the blood clears from the brain the haematomas break apart, contributing to the higher overall number of haematomas observed. A higher SA:V value equated to faster resolution. We repeated this analysis using stereomicroscopy and ImageJ to validate an alternative approach. Brightfield images of ICH+ larvae were acquired on the Leica snapshot and analysed to estimate haematoma area using ImageJ (Supplementary Fig. 1A). Brain regions were selected, and colour intensity was thresholded to red and area was determined (Supplementary Fig. 1B). A significant decrease in haemorrhage area between 2 and 3 dpf, and between 3 and 4 dpf was observed. Although haematoma size was variable at 4 dpf, no difference was found in haematoma area between 4 and 5 dpf.

To quantify the location of haematomas, groups of larvae exhibiting ICH were sacrificed at 2, 3, 4 and 5 dpf prior to protected status and stained for haemoglobin with *o*-dianisidine to visualize blood in the head (Fig. 1F). The number of larvae with visible haemorrhages decreased over time, with only 30% of larvae exhibiting haemoglobin staining at 5 dpf compared to 92% and 93% at 2 and 3 dpf, respectively (Fig. 1G). At 3 dpf, when the most haemorrhages were detected, haemorrhages were observed at higher frequencies in the hindbrain and forebrain (Fig. 1H). The prosencephalon contained 25.58% of the haemorrhages (11/43), 13.95% in the mesencephalon (6/43) and 39.53% in the rhomencephalon (17/43). Some bleeds were observed across multiple regions in the same larva, and 20.93% of haemorrhages (9/43) were observed in the ventricles.

### The majority of RBCs are cleared from the brain tissue into the ventricles

**Figure 2:**
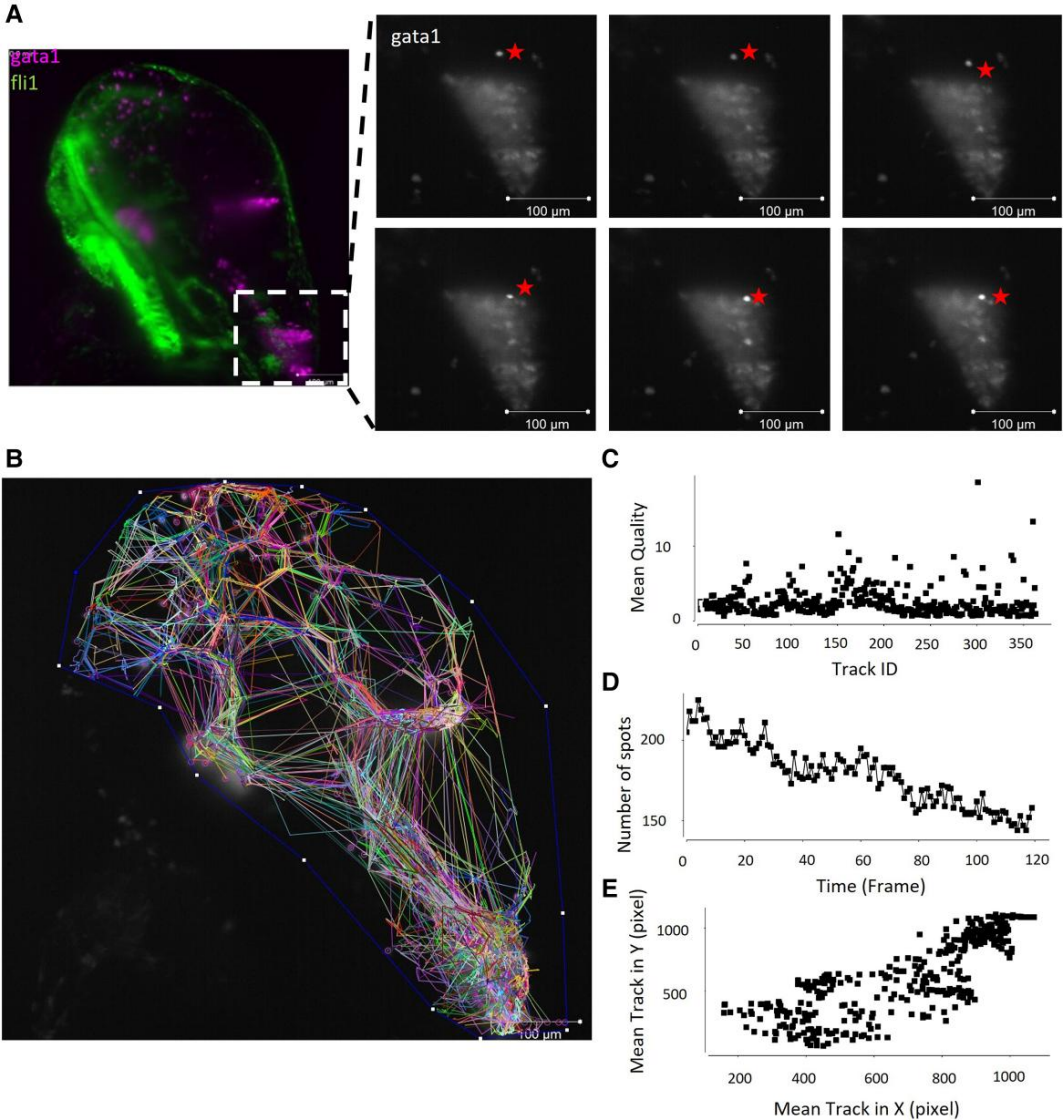
**Live time lapse imaging shows RBCs moving through the ventricular system.** A live fli1:EGFP;gata1:DsRed (endothelial cells, RBCs) transgenic larva were imaged over time to obtain a time lapse video (Video 1). (**A**) Still image from Video 1 (with false colour overlay) showing the location from the magnified insets. A gata1 positive RBC, denoted by a star, is observed in the hindbrain tissue and moves over the course of six frames (∼30 min) into the cluster of RBCs observed in the hindbrain ventricle. Scale bar = 100 µm. (**B**) Tracks of each gata1 positive cell over time show an overall migration to the hindbrain ventricle and movement within the haematoma. (**C**) The average automated detection quality was similar between the different cell tracks, one data point per cell. (**D**) Total number of cells identified (spots) decreased over the course of the time-lapse with the death of RBCs and the loss of gata1 fluorescence. (**E**) Average X movement versus average Y movement from each cell (spot) shows a high density of tracks migrating to the bottom right of the image, where the hindbrain ventricle is located.

Literature suggests erythrocytes may be cleared from the haematoma through active phagocytosis by macrophages/microglia, migration to the lymphatic system, or through lysis due to complement binding or metabolic depletion.^16^ To determine RBC lysis in mammals, T2*-weighted MRI scanning, detection of cytoplasmic proteins in peripheral blood, or haemoglobin detection in the peri-haematomal region can be used. These methods are not easily accessible in zebrafish larval models and so we utilized time-lapse fluorescent imaging to visualize the haematoma (gata1) over time at 78–84 hpf, a time point that aligns with haematoma clearance (Video 1). We observed the movement of gata1 positive RBCs from the tissue into the ventricles, where the haematoma is constantly moving (Fig. 2A). In Video 1, RBCs are observed moving in a forebrain–hindbrain direction along a central channel. No vascular endothelial cells (fli1 expression) were observed here, indicating that RBCs are not in circulation and may be flowing through the branching between ventricles.^30^ RBCs in the forebrain are cleared over the time-lapse imaging, with only 31% of cells still visible after 6 h (first frame to last). Overall gata1 fluorescence within the haematoma is lost gradually over the course of Video 1 and we attribute this to the cellular death of the erythrocytes and cells moving out of frame. Using Fiji TrackMate (v7.11.1),^31^ the migration (tracks) of gata1 positive cells (spots) was analysed over the course of Video 1 (Fig. 2B). Tracks generated by the algorithm show similar automated detection quality (Fig. 2C), and a steady decline in total number of spots tracked over time, probably due to the loss of individual particles into the haematoma and dying of cells with loss of fluorescence (Fig. 2D). Average X and Y spot movement (Fig. 2E) show a culmination to the bottom right of the image (maximum X and maximum Y) where the hind brain ventricle is. This observational data indicates that haematoma clearance quantified in Fig. 1 can be attributed to migration through the ventricular system.

### ICH-induced brain injury resolves spontaneously and is associated with an increase in nestin expression

**Figure 3:**
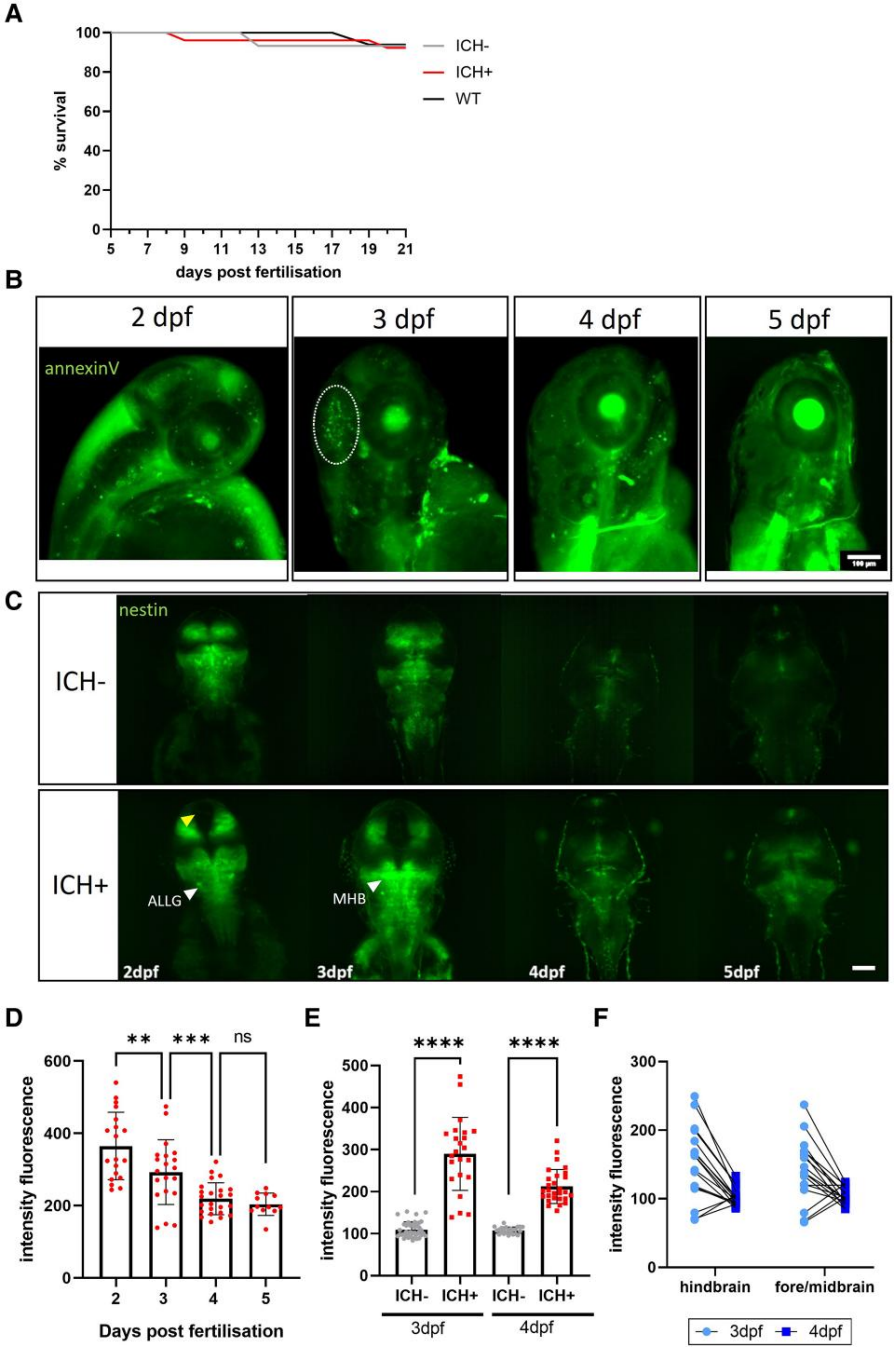
**The spontaneous recovery of brain injury after ICH is associated with increased nestin expression.** (**A**) Kaplan Meier analysis shows no difference in survival between WT, ICH− and ICH+ larvae at 21 dpf (93% ICH− and WT, 92% ICH+, *N* = 35 larvae per group at 5 dpf). (**B**) Representative images of annexinV expression in an ICH+ larvae at 2, 3, 4 and 5 dpf show apoptotic cells within the head during early development. A dense cluster of dying cells associated with ICH (denoted by white dotted line) at 3 dpf (24 h post-ICH) is resolved by 4 dpf. Scale bar = 100 µm. (**C**) Representative images of nestin positive larvae from both ICH− and ICH+ groups at 2–5 dpf. Global expression of nestin in the developing larval brain is lost over time with the greatest reduction in expression between 2 and 3 dpf. Overall, ICH+ larvae show a greater intensity of nestin expression. Yellow arrow denotes the absence of nestin expression between hemispheres and white arrows denote regions of intense expression of interest. Scale bar = 100 µm (*n* = 20, *n* = 12 at 5 dpf). (**D**) Quantification of the intensity fluorescence of nestin signal in the brain (*n* = 12–20). Data were analysed using a one-way ANOVA, **P = 0.0063, ***P = 0.0003, *F*(3,71) = 19.12. (**E**) Comparison of nestin expression in the brain at 3 and 4 dpf between ICH− and ICH+ larvae. Data were analysed using a one-way ANOVA ****P < 0.0001, *F*(3, 110) = 100.9. (**F**) Paired 3 and 4 dpf data show no difference in brain region of nestin expression. ICH−, intracerebral haemorrhage negative; ICH+, intracerebral haemorrhage positive; WT, wildtype; dpf, days post-fertilisation; ns, non-significant; ALLG, anterior lateral line ganglion; MHB, mid-hindbrain boundary.

It was noted that bbh homozygous larvae (ICH+) can be raised to adulthood for breeding and stock maintenance. A survival analysis confirmed that ICH+ larvae survive to early juvenile stages (up to 21 dpf) at the same frequency as ICH− siblings and WT larvae (Fig. 3A). We have previously shown in larvae treated with atorvastatin to initiate brain haemorrhages that brain cell injury, detected by annexinV signal in the head following ICH, resolves at 24 h post-ICH (Supplementary data in reference Crilly *et al*.^11^). Here, we showed the same recovery timeline in bbh larvae (Fig. 3B). The characteristic region of annexinV positive cells was present at 3 dpf, 24 h after ICH, however was resolved by 4 dpf. The recovery of dying cells in the brain aligns with a recovery in motility, as we have previously shown that spontaneous swimming time returns to baseline (ICH−) levels by 5 dpf in the bbh model.^11^

To investigate the role of NPCs in ICH recovery, we visualized nestin expression at the critical recovery time point between 3 and 4 dpf. Maximum intensity projections of lightsheet images show the expression pattern of the nestin positive cells in the brain at 2–5 dpf (Fig. 3C). Total expression was high at 2 and 3 dpf but overall intensity dropped at 4 dpf and expression was limited to specific proliferation zones^19^ (Fig. 3D). When compared to ICH− siblings, there was significantly higher intensity expression in ICH+ larvae at 3 and 4 dpf (Fig. 3E). To determine the differences in expression between brain regions (fore/midbrain and the hindbrain) images paired from 3 and 4 dpf were analysed and no significant differences were identified between brain regions (Fig. 3F). Data were also analysed for hemispheric differences and none were found. The mid-hindbrain boundary is a zone of continuous neurogenesis and contains the most nestin positive cells with the strongest nestin expression in ventricle contacting zones and ganglia.^19^ As the haemorrhages are so large and widespread across different brain regions, we measured overall nestin intensity across the whole brain. The brightest expression was in the mid-hindbrain boundary and along the midline. At 4 and 5 dpf in both ICH− and ICH+ larvae, expression was restricted to proliferative zones.

Notably, we observed a region absent of nestin expression between the two hemispheres of the midbrain in ICH+ larvae (yellow arrow, Fig. 3C), that is not present in the ICH− siblings. To determine whether this was a widening of the diencephalic ventricle, potentially due to haematoma and oedema, dextran (70 kDa) was injected into the hindbrain ventricle and imaged in ICH− and ICH+ larvae, however no differences were observed (Supplementary Fig. 2). There is no such space observed between hemispheres when larvae are treated with atorvastatin to initiate haemorrhage (Supplementary Fig. 3). We determined this to be unrelated to ICH, but could be an artefact of the bbh mutation, which has not been previously reported.

### Nestin positive cells are more proliferative and indicate oligodendrocyte differentiation following ICH

**Figure 4:**
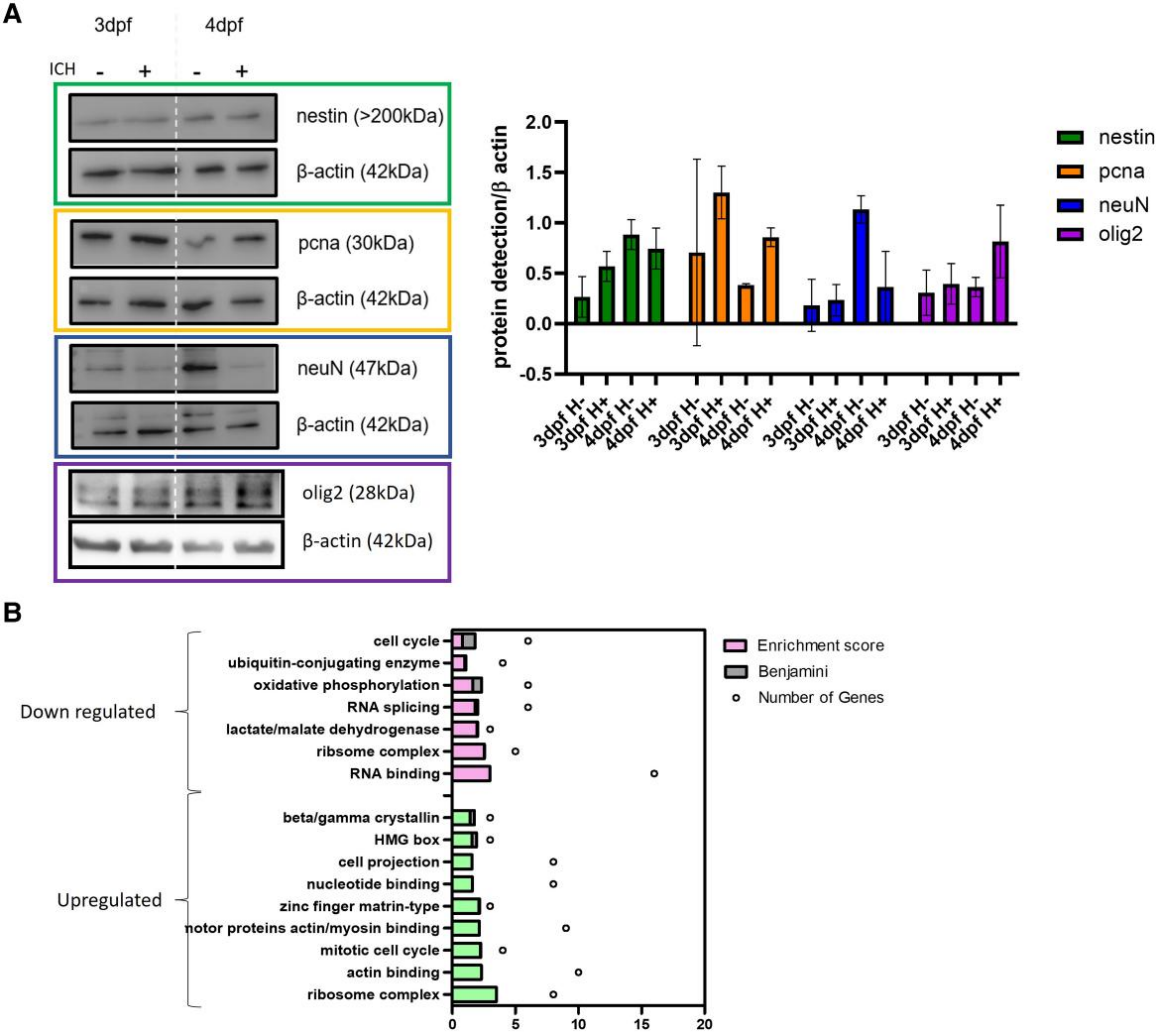
**Nestin positive cells are more proliferative in ICH+ than in ICH− siblings.** (**A**) Representative western blots and quantification analysis (*n* = 3) suggests that ICH+ larvae have less neuN*-*positive mature neurons than ICH− siblings, but pcna-positive proliferative cells and olig2-positive oligodendrocytes are increased in haemorrhage conditions (SD). Data analysed using a two-way ANOVA and no significant differences were found. Uncropped blots Supplementary Fig. 4. (**B**) Mass spec analysis of up (green) and down (pink) regulated pathways in ICH+ larvae between 3 and 4 dpf; dpf, days post-fertilisation.

We hypothesized that the NPCs may play a role in functional recovery after ICH due to increased expression at the critical time point of haematoma resolution and brain cell death recovery. To determine the cellular phenotype after injury, heads were removed, and lysates were used for western blot (Fig. 4A) (uncropped blots Supplementary Fig. 4). We detected more proliferating cell nuclear antigen (pcna) in ICH+ larvae at both 3 and 4 dpf, than in ICH− controls, indicative of a proliferative phenotype. We also observed that in ICH+ larvae at 4 dpf there was a decrease in neuronal nuclear protein (neuN) expression, a marker of mature, post-mitotic neurons and an increase in oligodendrocyte transcription factor (olig2) indicative of oligodendrocyte proliferation/differentiation. Oligodendrocytes have been identified as important for BBB integrity, increased angiogenesis, increased myelin production and alleviating infarct volume and brain swelling in ischaemic stroke.^32^

To confirm this proliferative phenotype, and to determine whether NPCs were differentiating into other neural cell types, we performed a proteomic analysis. Nestin positive cells were isolated from larval heads by FACS and mass spectrometry analysis on extracted peptides was performed. Data were acquired from four experimental groups (3 dpf ICH− and ICH+, 4 dpf ICH− and ICH+) and protein dysregulation was determined by ratios between ICH+ groups at 3 and 4 dpf. Processed data are openly available on figshare (DOI: 10.6084/m9.figshare.24981978). Quality control report available in Supplementary Fig. 5.

Proteins that were identified to be significantly dysregulated between control ICH− groups at 3 and 4 dpf were excluded from ontology analysis to determine the effect of haemorrhage only when compared between 3 dpf and 4 dpf. Results revealed 130 proteins significantly decreased and 95 proteins to be significantly increased including myelin basic protein (mbp) also indicative of oligodendrocyte differentiation. This was the only marker of oligodendrocytes identified as dysregulated in our dataset and other early markers of oligodendrocyte differentiation (PDGF, NG2, Olig1/2/3, OSP, MOG and SOX10) were not identified. This may be because if nestin positive NPCs were specialising to oligodendrocytes they would lose the nestin expression and therefore no longer be identifiable by FACS. Overall, pathways upregulated in 4dpf ICH+ compared to 3dpf ICH+ included cell cycle and protein synthesis pathways, indicating the increase of NPC proliferation (Fig. 4B). Other upregulated pathways associated with actin and myosin binding imply an increase in cell migration, supported by previous studies in rodents, which have found an increase in NPC migration to the site of damage following ICH in response to osteopontin.^33^

Pathways that were downregulated include the cell cycle and metabolic pathways such as oxidative phosphorylation, lactate dehydrogenase and RNA processing, which may also indicate further changes in cell division and protein synthesis. Beta/gamma crystallins are proteins normally abundant in the eye lens epithelial cells. Some studies suggest that crystallin β b2 (crybb2) has been associated with axonal elongation, promoting neurite lengthening through an autocrine mechanism.^34^

## Discussion

In this study, we sought to characterize the spontaneous recovery of zebrafish larvae from an ICH, to further validate the use of the model in the pre-protected stages before 5 dpf. We have shown that haematoma size is reduced and totally resolved in 70% of larvae by 5 dpf. Interestingly, we observed the clearance of RBCs into the ventricular system. The fish survive larval ICH and recover the functional deficits associated with injury, and therefore can be used for breeding. NPCs are upregulated in the immediate time after ICH and we speculate this may relate to oligodendrocyte differentiation, as a potential protective mechanism for the remyelination of neuronal cells after injury.

Haemorrhages are observed to first occur in bbh larvae at ∼33 hpf predominantly in the forebrain, however it appears that more blood is observed towards hindbrain regions at later time points. The bbh mutant brain haemorrhages occur due to weakened neovasculature and at this time-point key developing vessels in the hindbrain may be the cause of the rupture; namely the primordial hindbrain channel, the posterior cerebral vein and the mesencephalic vein.^35^ However, bbh larvae do not exhibit rebleeding,^27^ so it can be assumed that the increase in hindbrain blood at later time points is a consequence of RBCs migrating from the primary haematoma through the periventricular system, as oedema and CSF is cleared from the brain tissue. The evidence in this study suggests RBCs are moved in this way as the predominant mechanism of haematoma clearance in this model. It should be noted that although haematomas are observed to clear in all ICH+ larvae over this time period, it has only been visualized using time-lapse imaging in a single animal. As a result of this finding, a clinical question might be whether intraventricular haemorrhage results in faster haematoma clearance rates. It has previously been observed that RBCs also disappear when phagocytosed by macrophages/microglia and it would be interesting to determine what extent of clearance is dependent on these cell populations. Understanding the balance of these processes would be key to determining a regenerative therapy in this model and offer insights for translational studies.

In our model, the zebrafish resolve the haematoma and recover locomotor function deficits within 72 h of spontaneous ICH onset.^11^ Dependent on where the ICH is initiated in the mammalian brain, mice can take 14–21 days to recover functional deficits,^36^ rats 3 weeks^37^ and pigs 42 days.^38^ Clinically, humans can take up to three years to recover unassisted activities of daily life.^2^ The limitations with live imaging in mammals mean it is most common to determine haematoma size and resolution by MRI scanning and post-mortem analysis at restricted times and therefore difficult to build an accurate representation of blood resolution over time.

The nestin expression pattern in the forebrain was observed to differ in bbh ICH+ mutants compared to the heterozygous/wild-type siblings that did not exhibit haemorrhage, and to atorvastatin-induced ICH+ larvae. Rho-GTPases have been implicated in basic neuronal processes including proliferation, migration, plasticity and axon guidance and knocking out the protein function in mammals has been linked to neurodevelopmental conditions.^39,40^ It can be assumed that the changes in patterning may be due to the hypomorphic mutation in *βpix* (arhgef7); however, there were no gross morphological differences, or any differences observed in neuronal density (HuC:GFP expression, data not shown).

Myelination is important for repair and cellular protection after ICH.^41^ Oligodendrocytes are particularly susceptible to iron levels following ICH^42^ and their apoptosis contributes to the white matter damage after ICH through demyelination.^43^ Evidence in rat models of ICH shows that oligodendrocytes are indeed more proliferative following ICH as opposed to migratory,^41^ which is supported in this study. How ICH affects oligodendrocyte action and remyelination after injury is not well understood; for example it is unknown whether these highly proliferative cells can remyelinate damaged axons in the recovery period.^44^ This study may suggest that zebrafish are a suitable model for investigation into this early recovery period and cell-fate tracing experiments may reveal the nestin positive cell fate and interaction with the recovering/developing neurons. The data set is openly available on figshare (DOI: 10.6084/m9.figshare.24981978) to support further investigation across species and for translation, and direct research in non-regenerating species.

This study is an update on the use of the bubblehead zebrafish model for ICH investigation and it is imperative to provide the research community with this information, which may support use of the model in other capacities. The results from this study may support further investigation into the mechanistic role of oligodendrocytes in the immediate recovery period after ICH. Additionally, understanding how stem cells contribute to functional recovery after an ICH may reveal mechanisms that could be exploited as regenerative stem cell treatment strategies to encourage neural repair and functional rehabilitation in human patients.

## Supplementary Material

fcae310_Supplementary_Data

## Data Availability

All data can be requested from authors. Open data is available on figshare data repository DOI: 10.6084/m9.figshare.24981978.
