# Supplementary material for: Investigating recovery after a spontaneous intracerebral haemorrhage in zebrafish larvae
Source: Brain Commun. 2024 Sep 12;6(5):fcae310. doi: 10.1093/braincomms/fcae310 (PMC11483570; doi:10.1093/braincomms/fcae310)
Supplement: fcae310_Supplementary_Data [file fcae310_Supplementary_Data.docx]

Supplementary information


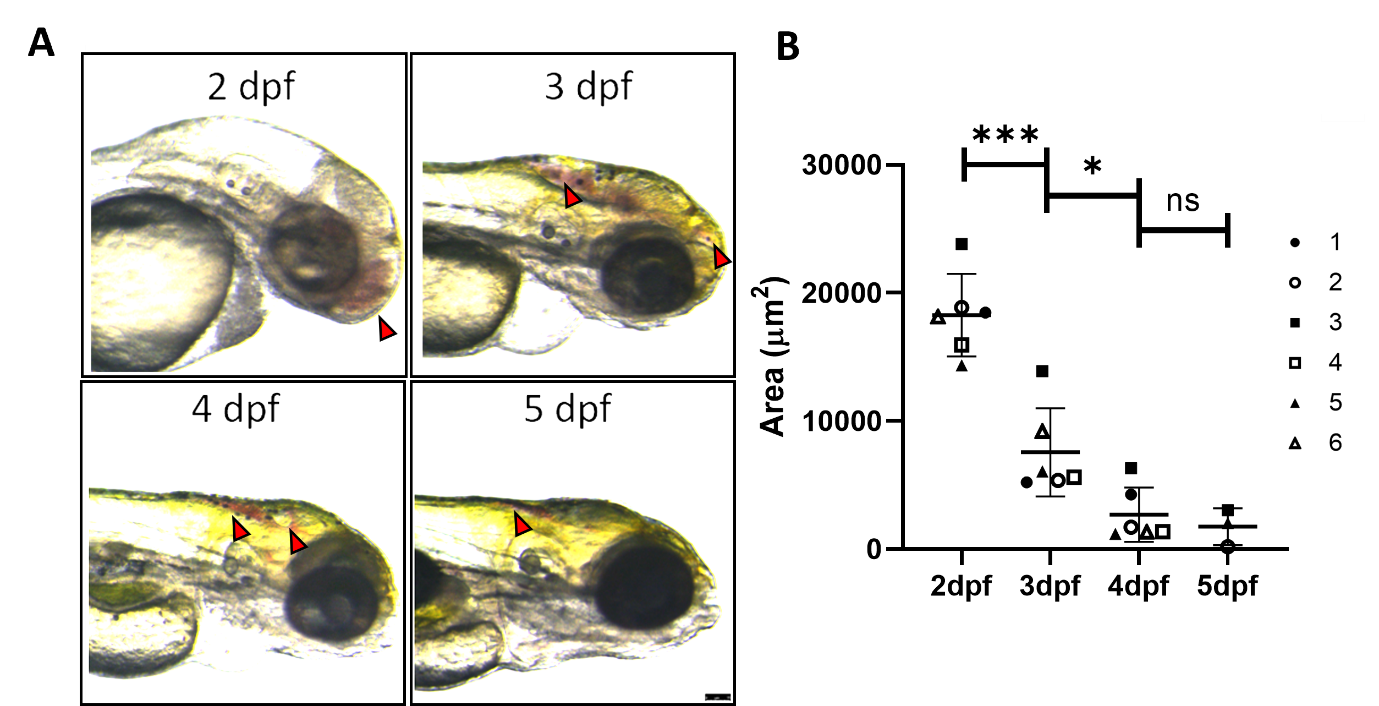


**Supplementary Figure 1** (A) Representative brightfield images of the bleed in a single larva at the onset of haemorrhage (2 dpf) and over the period of resolution (3-5 dpf). Arrows denote haemorrhages. Scale bars = 75 µm. (B) Quantification of red intensity in the brain from images acquired in (A). Symbol points pair time data for each individual. One-way ANOVA mixed-effects analysis (due to missing data points at 5 dpf) with Sidak’s post hoc shows significant differences between 2-3 dpf and 3-4 dpf. ***P=0.0002, *P=0.0175. F (1.517, 6.067) = 91.79.

**
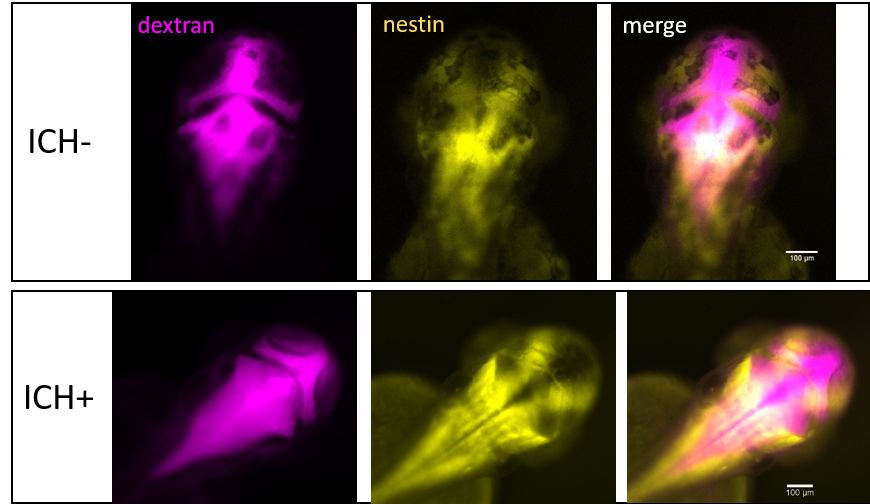
**

**Supplementary Figure 2:** Representative images of dextran (70 kDa) injections into the hind brain ventricle at 2 dpf shows the ventricular space in ICH- (top panels) and ICH+ (bottom panels) larvae to be the same (n=25). Scale bars = 100 µm.


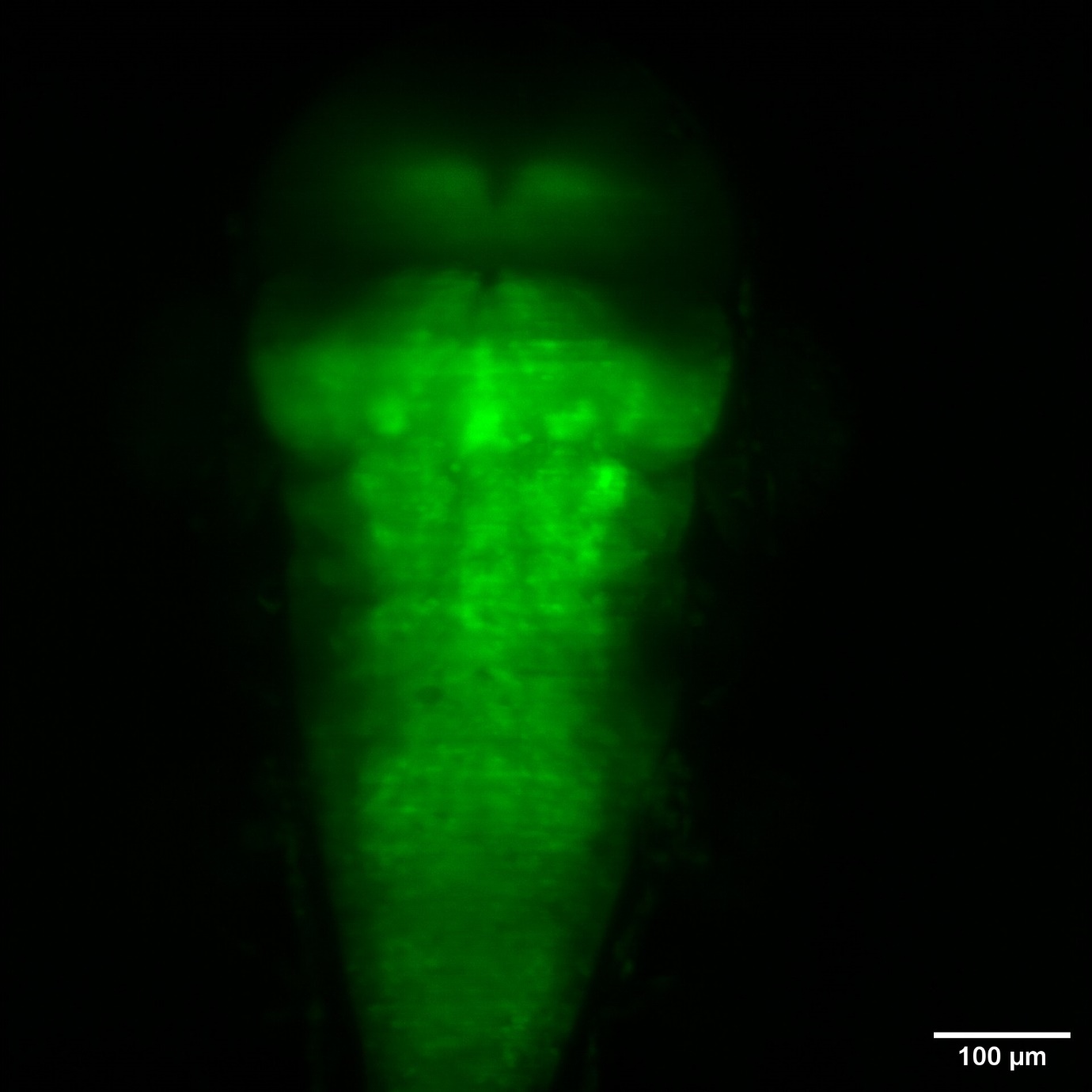


**Supplementary Figure 3**: Nestin expression in 2 dpf larvae treated with 1.0µM atorvastatin to induce haemorrhage.


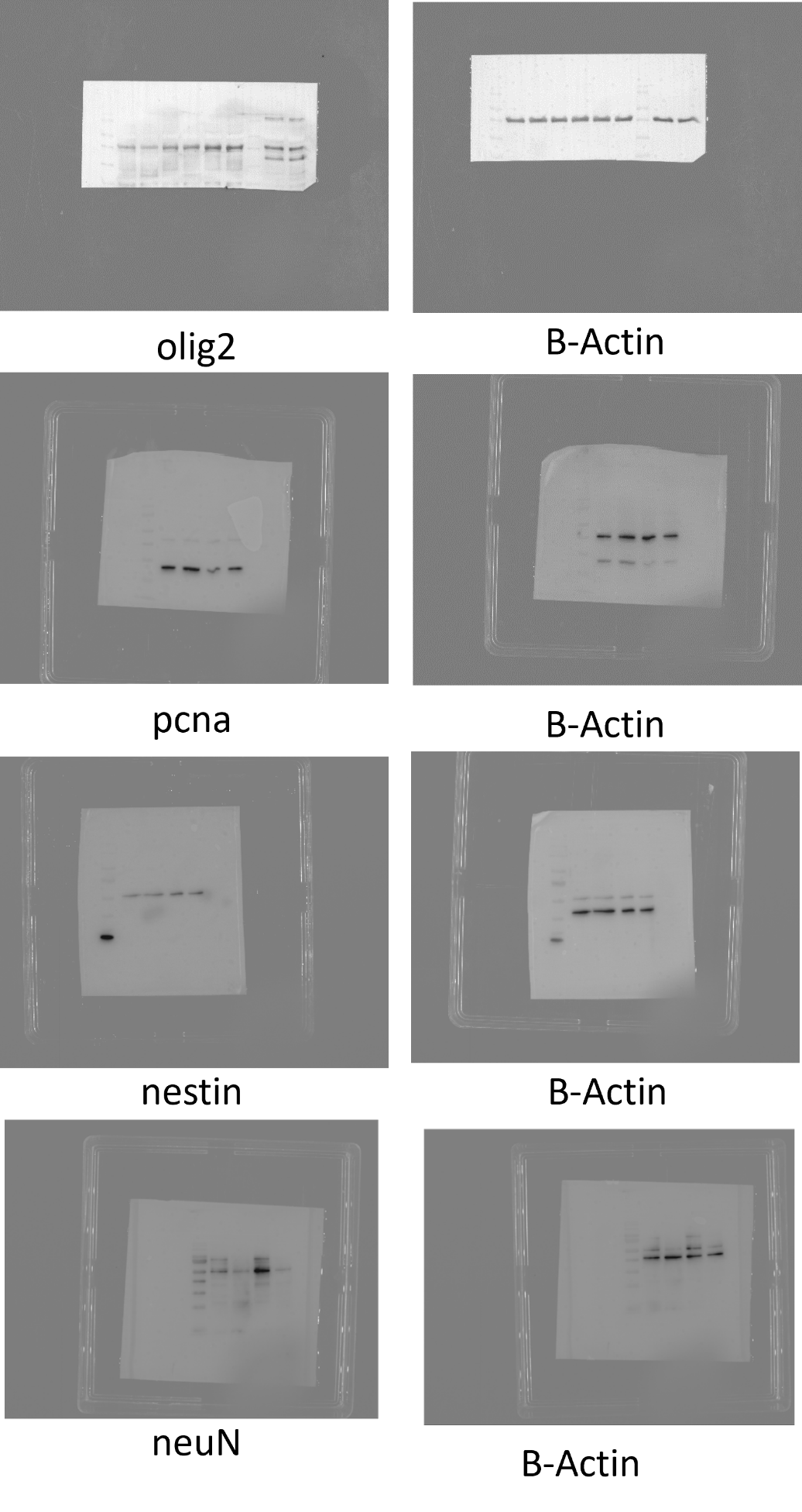


**Supplementary Figure 4**: Uncropped western blot images from Figure 4A


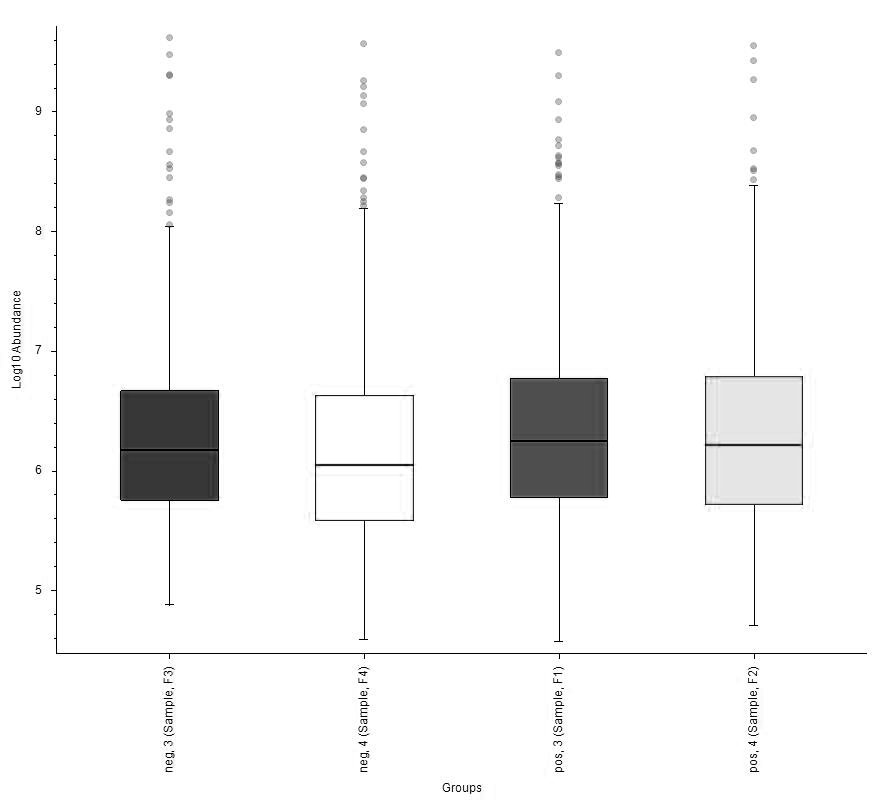

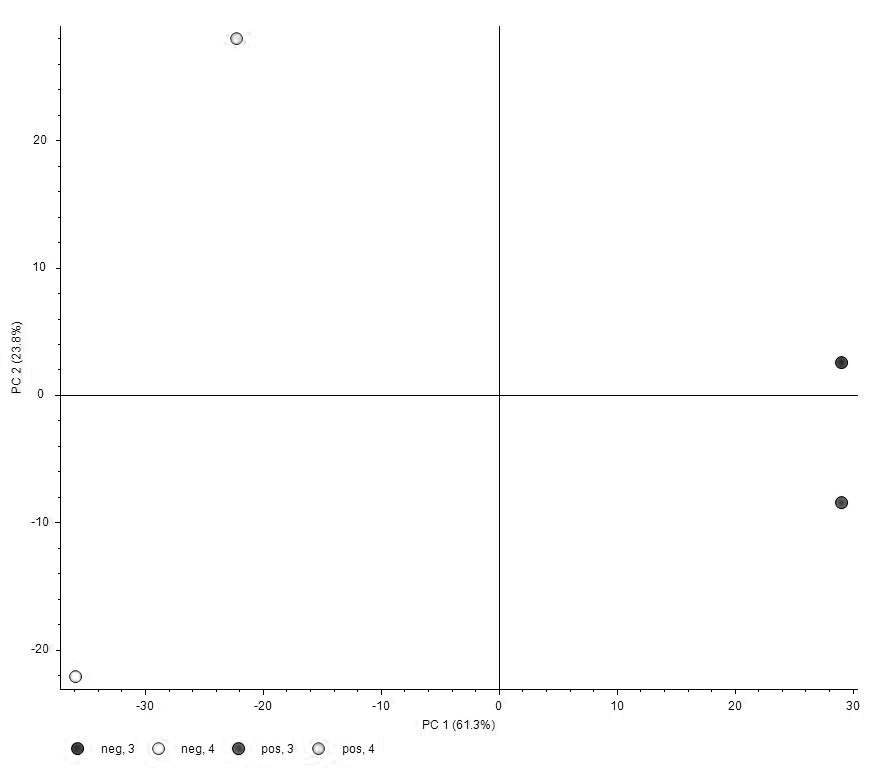


**Supplementary Figure 5**: QC figures from mass spectrometry show the principle component analysis (PCA), a visualisation of the variances, and normalised log10 abundances. Neg 3 = intracerebral haemorrhage negative, 3 days post fertilisation, neg 4 = intracerebral haemorrhage negative, 4 days post fertilisation, pos 3 = intracerebral haemorrhage positive, 3 days post fertilisation, pos 4 = intracerebral haemorrhage positive 4 days post fertilisation.
